# Draft Genome Sequence of Stenotrophomonas maltophilia KJ, a Clinical Isolate from Taiwan

**DOI:** 10.1128/mra.00058-22

**Published:** 2022-07-07

**Authors:** Li-Hua Li, Hsin-Hui Huang, Tsuey-Ching Yang, Chao-Jung Wu

**Affiliations:** a Department of Pathology and Laboratory Medicine, Taipei Veterans General Hospital, Taipei, Taiwan; b Ph.D. Program in Medical Biotechnology, Taipei Medical University, Taipei, Taiwan; c Department of Biotechnology and Laboratory Science in Medicine, National Yang Ming Chiao Tung University, Taipei, Taiwan; d School of Medical Laboratory Science and Biotechnology, College of Medical Science and Technology, Taipei Medical University, Taipei, Taiwan; Loyola University Chicago

## Abstract

We report the draft genome sequence of Stenotrophomonas maltophilia strain KJ, which was isolated from a sputum sample from a patient with a respiratory tract infection. Multilocus sequence typing analysis suggested that strain KJ belongs to a novel S. maltophilia sequence type.

## ANNOUNCEMENT

Stenotrophomonas maltophilia, a Gram-negative bacterium, is ubiquitous in the environment, including water, soil, plants, and hospitals (1). S. maltophilia is drawing attention because it can cause pneumonia and bacteremia in immunocompromised patients (2). This bacterium harbors multiple antimicrobial resistance determinants (ARDs) and is naturally resistant to classes of antibiotics, including β-lactams, aminoglycosides, and macrolides, which limits the treatment options for S. maltophilia infections (2).

S. maltophilia KJ, a clinical isolate, was isolated from a sputum sample from a patient with a respiratory tract infection. Bacteria were cultured on blood agar at 37°C in ambient air. The species identification was performed using the ID32 GN system (bioMérieux, France), followed by 16S rRNA PCR confirmation (3). The genomic DNA was extracted using the DNeasy extraction kit (Qiagen, Germany), and the sequencing library was constructed using the Nextera DNA Flex library preparation kit (Illumina, USA). The sequencing run was performed on an Illumina MiSeq system in 150-bp paired-end sequencing mode. A total of 99 Mb of raw data with 492,500 paired-end reads was generated from the sequencing run. Trim Galore v0.5.0 (https://github.com/FelixKrueger/TrimGalore) was applied to remove low-quality reads (Phred scores of ≤20) and to trim Illumina adaptors. The genome was assembled with SPAdes v3.12.0 (4) in the careful mode, and contigs of <500 bp were removed. Genome coverage of 40× was reported. The draft genome size of KJ was 4,879,596 bp, containing 70 contigs with a GC content of 65%. The *N*_50_ value of the contigs was 182,983 bp. The assembled genome was annotated by the NCBI Prokaryotic Genome Annotation Pipeline (PGAP) v5.3 (5), and 4,580 genes, 4,505 coding sequences (CDSs), 5 complete rRNAs, 66 tRNAs, 4 noncoding RNAs, and 30 pseudogenes were revealed. PHASTER (https://phaster.ca) was applied to identify prophage sequences, and a prophage of size 36.6 kbp and GC content of 63.39% was predicted for contig_00001 (6). Multilocus sequence typing (MLST) analysis was performed by querying the genome sequence against the PubMLST Stenotrophomonas maltophilia typing database (https://pubmlst.org) (7). The results showed that isolate KJ had alleles 4, 5, 70, 6, and 85 in loci *atpD*, *mutM*, *nuoD*, *ppsA*, and *recA*, respectively. The *gapA* and *guaA* genes of KJ were predicted in contigs 1 and 17, respectively. However, no exact allele matches for loci *gapA* and *guaA* were found in the PubMLST database (7), suggesting that KJ harbors a novel S. maltophilia sequence type.

The genomes of strains KJ and K279a (GenBank accession number NC_010943) were compared using Roary v3.13.0 (8). With a cutoff value of ≥95% protein identity, 3,508 CDSs were present in both genomes and at least 947 CDSs were uniquely harbored by strain KJ. Among the exclusive CDSs of strain KJ, four gene clusters attracted our attention, that is, LAW54_01165-LAW54_01205, LAW54_01300-LAW54_01320, LAW54_02885-LAW54_2925, and LAW54_02940-LAW54_2960. The proteins encoded by the four gene clusters seem to be responsible for conjugation, suggesting the superiority of KJ in the horizontal transfer of genetic elements. In addition, a gene cluster (LAW54_15515-LAW54_15555) whose encoded proteins appear to be involved in cellulose biosynthesis was present in strain KJ but absent in strain K279a, suggesting that KJ is a potential strain for biotechnology such as food production, cosmetics, and even medical applications (9).

### Data availability.

The assembled KJ genome sequence was deposited in DDBJ/EMBL/GenBank with accession number JAIQXD000000000. The raw sequence reads are available in the SRA (accession number SRR15802036) under BioProject accession number PRJNA761510.
